# The neural basis of monitoring goal progress

**DOI:** 10.3389/fnhum.2014.00688

**Published:** 2014-09-10

**Authors:** Yael Benn, Thomas L. Webb, Betty P. I. Chang, Yu-Hsuan Sun, Iain D. Wilkinson, Tom F. D. Farrow

**Affiliations:** ^1^Department of Psychology, University of SheffieldSheffield, UK; ^2^Academic Unit of Radiology, Royal Hallamshire Hospital University of SheffieldSheffield, UK; ^3^Academic Clinical Neurology, Department of Neuroscience, University of SheffieldSheffield, UK

**Keywords:** progress monitoring, conflict monitoring, error monitoring, self-regulation, dorsal anterior cingulate cortex, cuneus

## Abstract

The neural basis of progress monitoring has received relatively little attention compared to other sub-processes that are involved in goal directed behavior such as motor control and response inhibition. Studies of error-monitoring have identified the dorsal anterior cingulate cortex (dACC) as a structure that is sensitive to conflict detection, and triggers corrective action. However, monitoring goal progress involves monitoring correct as well as erroneous events over a period of time. In the present research, 20 healthy participants underwent functional magnetic resonance imagining (fMRI) while playing a game that involved monitoring progress toward either a numerical or a visuo-spatial target. The findings confirmed the role of the dACC in detecting situations in which the current state may conflict with the desired state, but also revealed activations in the frontal and parietal regions, pointing to the involvement of processes such as attention and working memory (WM) in monitoring progress over time. In addition, activation of the cuneus was associated with monitoring progress toward a specific target presented in the visual modality. This is the first time that activation in this region has been linked to higher-order processing of goal-relevant information, rather than low-level anticipation of visual stimuli. Taken together, these findings identify the neural substrates involved in monitoring progress over time, and how these extend beyond activations observed in conflict and error monitoring.

## Introduction

The majority of human activity is goal-directed (Locke, 1969) and consequently, the process by which people strive for goals has been the focus of much social, cognitive, and neuropsychological research. Berkman and Lieberman (2009) drew on dominant frameworks for understanding goal-directed behavior (e.g., Miller et al., 1960; Wicklund and Gollwitzer, 1981; Kuhl, 1984; Gollwitzer, 1990; Higgins et al., 1994; Carver and Scheier, 1998) to suggest that goal striving can be divided into several sub-processes, namely goal setting, attention, motor control, response inhibition, and progress monitoring. For example, if a person sets himself the goal to lose weight, he may start to pay attention to information related to losing weight (such as the caloric value of food), increase his level of physical activity, inhibit the urge to eat sugary snacks, and periodically weigh himself to check whether progress is being made. It is generally agreed that all of these processes are important for successful goal achievement (e.g., Carver and Scheier, 1990, 1998; Higgins, 1996). However, while the neural bases of attention, motor control, and response inhibition have received considerable research attention (for a review, see Berkman and Lieberman, 2009), less research has focused on the neural basis of progress monitoring (Liberman and Dar, 2009), despite indications that monitoring may be essential for processes such as inhibitory control (Chatham et al., 2012). Furthermore, research that does purport to investigate progress monitoring has tended to focus on the neural systems involved in the detection of errors, or situations where errors are likely to occur.

Progress monitoring, however, involves more than simply the detection of errors, and reviews (e.g., Carver and Scheier, 1990; Webb et al., 2013) point to the importance of four processes: (i) periodically attending to relevant information (e.g., the caloric value of food items, or weight as indicated by a set of scales), (ii) updating the current state in working memory (WM) (e.g., replacing previous weight with current weight); (iii) comparing the information to a target or reference value (e.g., current weight compared to desired weight); and (iv) detecting a discrepancy (e.g., that current weight is higher than expected). The processes of comparing information on the current state with a target or reference value and detecting a discrepancy have been often studied using three main tasks: the Stroop task (e.g., Pardo et al., 1990), the flanker task (e.g., Ullsperger and von Cramon, 2001), and the Simon task (e.g., Kerns, 2006). However, comparing information to a reference value does not always result in the identification of a discrepancy (e.g., if progress is going as expected), and thus should be considered as a separate process from the detection of errors. Furthermore, some researchers (e.g., Berkman and Lieberman, 2009) include the selection of actions designed to reduce discrepancy (e.g., changing one's diet) as part of the process of progress monitoring. However, while action selection may be essential for successful goal-directed behavior, it is not part of the monitoring process, as monitoring may not always result in action, or indicate that action is required. There is, therefore, a need to rethink the way that the neural basis of progress monitoring has been examined to date.

### Current evidence on the neural basis of progress monitoring

A range of evidence points to the likely neural basis of processes involved in progress monitoring (for a review, see Berkman and Lieberman, 2009). In the following, we focus on the roles of attention, WM, and the detection and assessment of discrepancies between current and desired states.

#### Attention

Attention is likely to be the first process that is engaged when monitoring goal progress. Attention is defined as the process that selects which sensory information is processed (and possibly reaches awareness) at any one time. As such, attention is considered to be a multi-channel process that can be driven by either bottom–up (e.g., when attention is diverted to a salient stimulus) or top–down (e.g., goal directed) processes. While stimulus-driven attention is mostly supported by the ventral network (that consists of the lateral and inferior frontal/prefrontal cortex (PFC) and the temporo-parietal junction, Corbetta and Shulman, 2002], the dorsal network [that includes the frontal eye field; Brodmann Area (BA)8] and the superior parietal cortex have been shown to be involved in goal-directed attention (Corbetta and Shulman, 2002; Fox et al., 2005).

How bottom–up and top–down systems interact to control attention has been the focus of considerable research in recent years (e.g., Buschman and Miller, 2007; Asplund et al., 2010). For example, Spreng et al. (2013), examined connectivity within and between the neural networks supporting top–down and bottom–up attention. They suggest that, while each of the two networks are mostly connected within themselves, a third fronto-parietal network (Vincent et al., 2008; Niendam et al., 2012) flexibly engages with either of the networks (Spreng et al., 2010; Spreng and Schacter, 2012). This fronto-parietal “control” network includes the lateral prefrontal cortex, precuneus, inferior parietal lobule, medial superior prefrontal cortex, and the insula (Vincent et al., 2008; Spreng et al., 2010; Niendam et al., 2012). It has been suggested that this fronto-parietal network plays an important role in goal-directed behavior, as it flexibly directs the focus of attention from one network to another, and hence provides “executive control” of directed attention. It therefore seems likely that this network is involved in monitoring progress toward a current goal.

Finally, there is some evidence that top–down directed attention can modulate activity in early sensory cortices, resulting in regions such as the primary visual cortex, that are often associated with processing of existing visual stimuli, being activated in anticipation of a specific visual stimulus (Chawla et al., 1999; Kastner et al., 1999; Hopfinger et al., 2000). In relation to monitoring goal progress, therefore, this finding suggests that goal-related information, particularly if anticipated, is more likely to be attended to. This idea is consistent with research which suggests that people are “perceptually ready” to encounter stimuli that are relevant for attaining their goal (Aarts et al., 2001).

#### Working memory

Attending to information is the first step involved in progress monitoring. However, this information then needs to be stored and updated as new information becomes available. Storage and updating is considered to involve WM (Baddeley, 2010). Similar to attentional processes, research has suggested that WM is supported by a network of brain regions that extends from the PFC to the parietal cortex (Gazzaley et al., 2004; Sauseng et al., 2005). For example, it was recently shown that patients with schizophrenia, who show WM deficits, also exhibit reduced connectivity in the fronto-parietal network. This extensive fronto-parietal network has distinguishable roles: while the posterior parietal region is involved in manipulating information, the dorso-lateral PFC (DLPFC) is associated with keeping track of the information that is being manipulated (Champod and Petrides, 2007, 2010). Given that monitoring goal progress may involve manipulating information (e.g., to allow comparison with relevant reference values, Ashford and Cummings, 1983; or to emphasize certain aspects, Huang et al., 2012) both the posterior parietal region and the DLPFC are likely to be involved.

Behaviorally and from a neuroscience perspective, there seem to be close relationships, and possibly an overlap, between processes involved in tasks that engage WM and those that involve selective attention (Gazzaley and Nobre, 2012). Behaviorally, selective attention has been shown to enhance WM performance (Murray et al., 2011; Backer and Alain, 2012). From a neuroscience perspective, repetitive transcranial magnetic stimulation directed at the inferior frontal junction, which has previously shown to be involved in modulating visual attention (Zanto et al., 2010), reduces the accuracy of WM (Zanto et al., 2011). These findings suggest that processes involved in WM and attention may be difficult to distinguish from one another. In the context of progress monitoring, it means that attending to information, committing it to memory, and updating the information as new information becomes available, are likely to be difficult to empirically distinguish. While research to date has attempted to understand these processes in isolation, there may be added value in observing how these processes interact in a relatively naturalistic task such as monitoring progress over a period of time.

#### Detection and assessment of discrepancies

Once the current state has been identified and committed to memory, the person seeking to evaluate their goal progress must compare that information to their reference value or goal and identify any discrepancies. Most research to date has investigated the neural basis of discrepancy detection (i.e., recognizing that the current state does not match the reference value) using paradigms involving error detection (i.e., identifying conflict between the current and desired response). For example, the first study using functional magnetic resonance imagining (fMRI) to examine error monitoring (Carter et al., 1998) used a Continuous Performance Test, in which participants were presented with probes (either A or B) preceded by cues (either X or Y). The target response was only to be performed when the letter A was followed by the letter X. The results suggested that the anterior cingulate cortex (ACC) was active when participants made an error. However, Carter and colleagues also observed activation in the ACC when participants responded correctly if there was increased response competition (i.e., BX and AY, which has one letter correct, compared with AX or BY where both letters are either correct or incorrect). Carter and colleagues concluded that the ACC is involved in detecting conditions under which errors are *likely* to occur (i.e., detecting potential conflict) rather than being activated in response to the errors themselves, and hence is used to detect situations in which action (or inhibition) is required (for a review, see Botvinick et al., 2001).

Since Carter et al.'s study, similar paradigms have been studied using a range of neuroimaging techniques, including intracranial recordings (Gehring et al., 1993; Brázdil et al., 2002, 2005; Wang et al., 2005; Pourtois et al., 2010), magnetoencaphalography (MEG) (Miltner et al., 2003; Keil et al., 2010), and fMRI (Holroyd et al., 2004; Debener et al., 2005). More recently, it has been suggested that the ACC's structural and functional organization can be subdivided (Vogt, 2009). One of its sub-structures—the dorsal ACC (dACC)—has been reported to be involved in the detection of conflict between, for example, intended and actual responses (Berkman et al., 2012, for a review, see Carter and Van Veen, 2007). Given that the detection of errors and situations in which erroneous responses are likely is one aspect of monitoring goal progress over time, it is reasonable to expect that the dACC will be involved in monitoring goal progress.

### Unanswered conceptual and methodological issues

Despite a range of evidence on the likely neural regions involved in monitoring goal progress, several conceptual and methodological issues remain unanswered. Monitoring goal progress is a complex process that simultaneously involves elements or combinations of the processes described above. That is to say, while these sub-processes are temporally organized, they are not easily distinguishable (e.g., WM and attention overlap, as discussed above), and processes are not necessarily evoked in a linear temporal fashion. For example, Control Theory (Carver and Scheier, 1982, 1990) suggests that attending to information on goal progress identifies the current state (e.g., current weight), which is then compared to the desired state, and any discrepancy is identified and used to inform subsequent goal-directed effort. Progress monitoring (and its associated sub-processes) are therefore part of a continuous feedback loop, rather than a linear temporal sequence. However, despite the dynamic and continuous nature of progress monitoring, most studies to date examine the sub-processes of progress monitoring in isolation, often employing an event-related design. Studying the sub-processes of goal-directed behavior in isolation may limit our understanding of how these processes are integrated and operate in real-life.

In an effort to overcome this problem, Berkman et al. (2012) used a mixed design. However, their study still employed a task that only required participants to monitor their performance on each trial separately. As a result, it is difficult to know whether the same neural regions, such as those involved in WM or sustained and directed attention, would be involved if participants were required to monitor their progress over a series of trials. Furthermore, the variant of the go-no go task used by Berkman et al. (2012) still involved contrasting erroneous with correct responses, raising the possibility that some elements of progress monitoring, such as those involved in comparing the current state to the desired outcome (a process that would be invoked even if the response were correct), were not examined.

There are a number of other limitations to studying the neural basis of progress monitoring using tasks that only involve trial-by-trial monitoring. Typically, monitoring goal progress requires that the person periodically attend to relevant information (Berger, 2002). This may take place over a period of minutes (e.g., achieving a target in a game) or days (e.g., assessing progress toward the goal of losing weight before the summer). Berkman and Lieberman (2009) point to the need to develop tasks that reflect, within the constraints of neuroimaging studies, monitoring over the medium term, stating that: “Neuroscientists have yet to examine long-term goals. Although methodological constraints limit our ability to examine the representation of long-term goals in a magnetic resonance imaging (MRI) scanner (e.g., imaging participants while they engage in the task of being a ‘good American’), an initial step would be to extend our existing knowledge of short-term goals to slightly broader ones, termed here ‘medium-term’ goals.” (p. 104). Therefore, there is a need to study progress monitoring using a block-rather than an event-related design, in order to examine how the sub-processes of progress monitoring, that have until now been explored individually, are integrated when people assess their progress on tasks that span over time and multiple events.

Finally, as noted earlier, previous studies have focused on conflict monitoring, and have tended to employ paradigms involving error detection. However, monitoring goal progress is fundamentally different to monitoring for errors, as progress monitoring is an ongoing process where relevant information has to be updated, or aggregated, in order to assess the current state in relation to the goal. In contrast, error monitoring involves identifying discrete errors. Furthermore, while conflict monitoring focuses on detecting errors, or detecting situations where errors may occur, progress monitoring involves both the detection of erroneous and correct responses (i.e., in both instances people ask themselves “did I get that one right?”). Therefore, focusing on the brain regions activated by erroneous, compared with correct, responses is likely to mask the areas that are involved in progress monitoring.

### The present research

The present research investigated the neural basis of monitoring progress over a medium term (rather than on a trial-by-trial basis) in a context that does not rely on comparing the neural substrates of erroneous responses to correct ones. Two computer games were designed whose conditions differed in the nature of the progress monitoring that was required. One computer game involved processing numerical stimuli, since progress monitoring often involves numerical processing (e.g., checking finances, counting calories, etc.) and evidence suggests that people find it easier to monitor quantifiable outcomes (Josephs et al., 1994; Chang et al., 2013). The other game involved monitoring progress toward the goal of recreating a pattern of shapes; a task that relied on visuo-spatial processing. Both tasks were designed not to give participants direct feedback on the to-be-monitored aspect of the task, but rather to examine self-initiated progress monitoring by asking participants to keep track of the relevant dimension. Each of the tasks were designed to minimize individual variations in the process of monitoring (e.g., by providing specific instructions), allowing the contrasts between conditions to reveal the neural substrates that are involved in the various processes involved in monitoring goal progress.

Given the role of the parietal cortex in selective attention and WM (Gazzaley and Nobre, 2012) and in processing visuo-spatial (Culham and Kanwisher, 2001) and numerical stimuli (Dehaene et al., 2003), we hypothesized that monitoring progress in the present study would activate a core network in the parietal cortex that is common to both numerical and visuo-spatial tasks (Benn et al., 2012). Specifically, we anticipated that monitoring progress over time (as compared to trial-by-trial monitoring, and the baseline conditions) would involve bilateral parietal activation. Furthermore, we expected to observe activation of the fronto-parietal network, due to the task demanding attention and WM resources (Gazzaley and Nobre, 2012). Specifically, we anticipated activation in the DLPFC due to its role in updating WM (Petrides, 2000; Provost et al., 2010), which is an important part of monitoring progress over time (e.g., to carry over the current value from one trial to the next). Lastly, we hypothesized that we would observe activation in the dACC when participants were monitoring their progress toward a specific reference value, and hence when there is the greatest potential for a discrepancy to occur between desired and actual responses. However, activation of other areas within the cingulate gyrus was also hypothesized, due to its reported role in many tasks requiring attention and executive control (Fan et al., 2005).

## Methods

### Ethical statement

Ethical approval for the study was granted by the Ethics Sub-Committee in the Department of Psychology at The University of Sheffield. The research was conducted in a manner consistent with the American Psychological Association's ethical principles.

### Participants

Twenty healthy, native English speaking, right-handed (as assessed by the revised Edinburgh handedness questionnaire, Oldfield, 1971; Cohen, 2008) undergraduate students (Mean age = 18.90 years, *SD* = 0.31) received £40 for participating in the study. Half of the participants were female and all had normal or corrected-to-normal vision, and were screened for MRI compatibility. Prior to the study, participants were informed that the study investigated the neural correlates of computer game playing. At the end of the study, participants were debriefed about the real purpose of the study, and were given the chance to ask any questions.

### Stimuli and design

**Figure 1:**
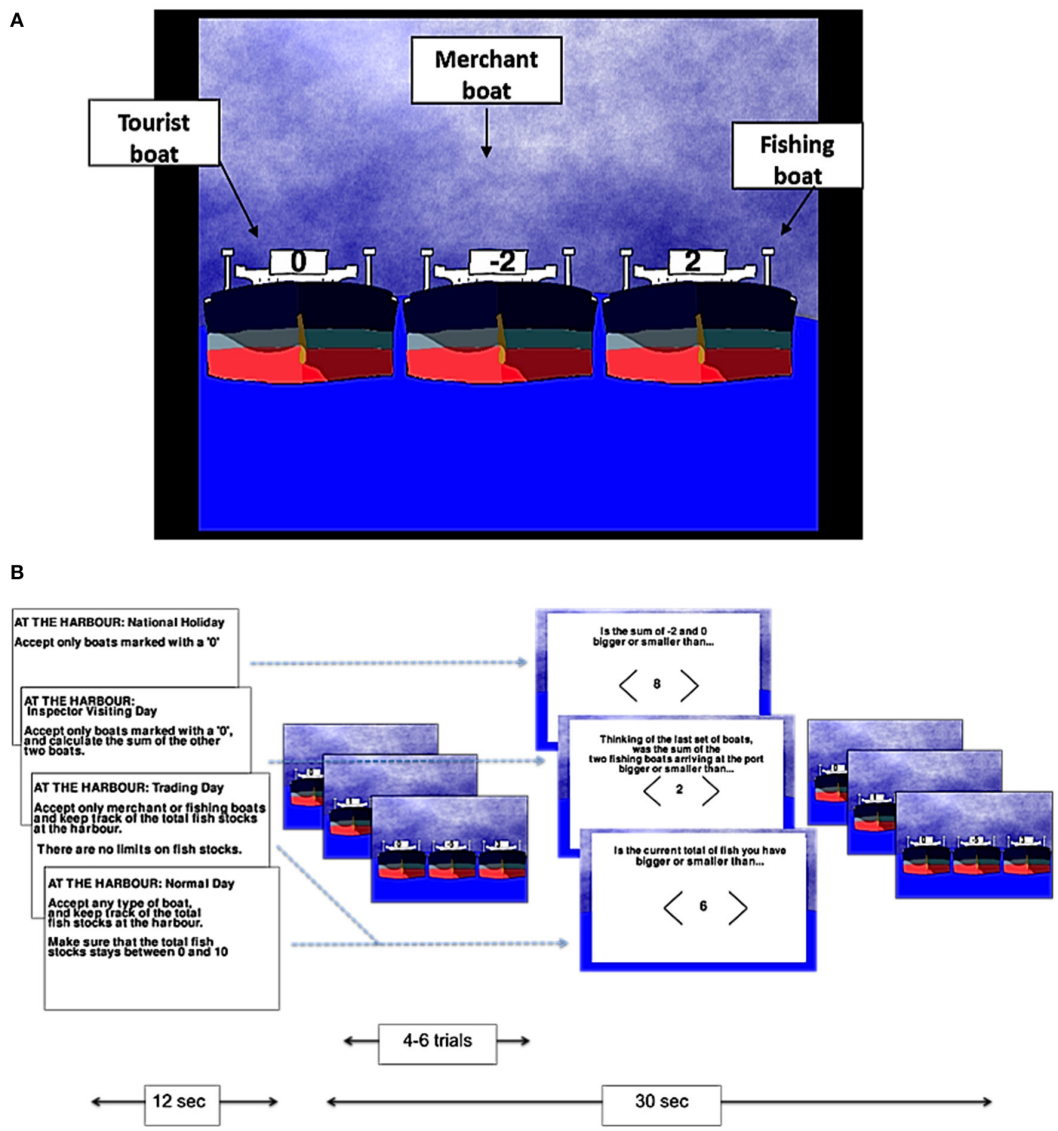
**The harbormaster game**. Illustration of the type of boats **(A)** and experimental procedure **(B)**.

The tasks were developed using Pygame 1.9.1. Participants played two different games: a numerical game termed the “harbormaster game” and a visuo-spatial game termed the “nursery game.” In the harbormaster game, participants were asked to play the role of a harbormaster at a busy port. As such, participants were presented with three boats arriving at the port on each trial, and asked to decide which of the three boats should enter the port (by selecting a boat using an MR-compatible mouse; NAtA technologies, FOM-2B-10B fMRI Mouse). There were three different types of boats indicated by different values; boats marked with a zero represented tourist boats (no fish bought or sold), boats with a positive number represented fishing boats (bringing fish into the port, with the amount of fish indicated by the number on the boat), and boats with a negative number represented merchant boats (wishing to buy the amount of fish indicated by the number on the boat; Figure 1A).

There were four conditions in the harbormaster game: In the *no monitoring condition* (Condition 1), participants were told that it was a national holiday and, as such, only tourist boats should be allowed to enter the port. Condition 1 was used as a control condition to subtract the elements of calculation, visual processing, and motor responses from the subsequent conditions. In the *trial-by-trial monitoring condition* (Condition 2), participants were also instructed to only allow tourist boats to enter the port. However, in addition, they were also told that an inspector may visit the port and that they would need to calculate the sum of fish arriving at the port on each trial, ready to report to the inspector. As such, participants were required to select the tourist boat, and to calculate the sum of the other two boats separately on each trial (without having to carry it over from one trial to the next). Condition 2, therefore, required trial-by-trial monitoring, and enabled us to differentiate this process from monitoring over time.

In the condition involving *monitoring information over time without a reference value* (Condition 3), participants were told that it was a trading day and, as such, they were instructed to only allow merchant or fishing boats to enter the port and to keep track of the cumulative total of fish held at the port over the trials. Participants in this condition were not given a clear target, but were rather told to “maximize trading.” Hence, Condition 3 allowed us to examine the processes involved in monitoring progress over time, but without comparing the current state to a specific reference value. Finally, in the condition involving *monitoring over time with respect to a reference value* (Condition 4), participants were told that they could allow any boat to enter the port in each trial, but that they should ensure that cumulative fish stocks at the port did not go below zero or above a randomly set number between four and 10 (depending on the storage capacity at the port that day), set at the start of the condition. Condition 4, therefore, enabled us to examine the neural basis of monitoring over time with respect to a reference value.

In each of the conditions, after every 4–6 trials, participants were asked a question to test whether they had followed the instructions. In Conditions 3 and 4, participants were asked about the current fish stocks at the port. In Condition 2, they were asked to report the sum of the two non-zero boats that had arrived at the port on the last trial. In Condition 1, participants were given a simple calculation question, similar to the one required in Condition 2, but unrelated to the task (e.g., the sum of −2 and 0, as illustrated in Figure 1B). In all conditions, participants used the mouse to indicate whether their answer was larger or smaller than a number shown on the screen.

**Figure 2:**
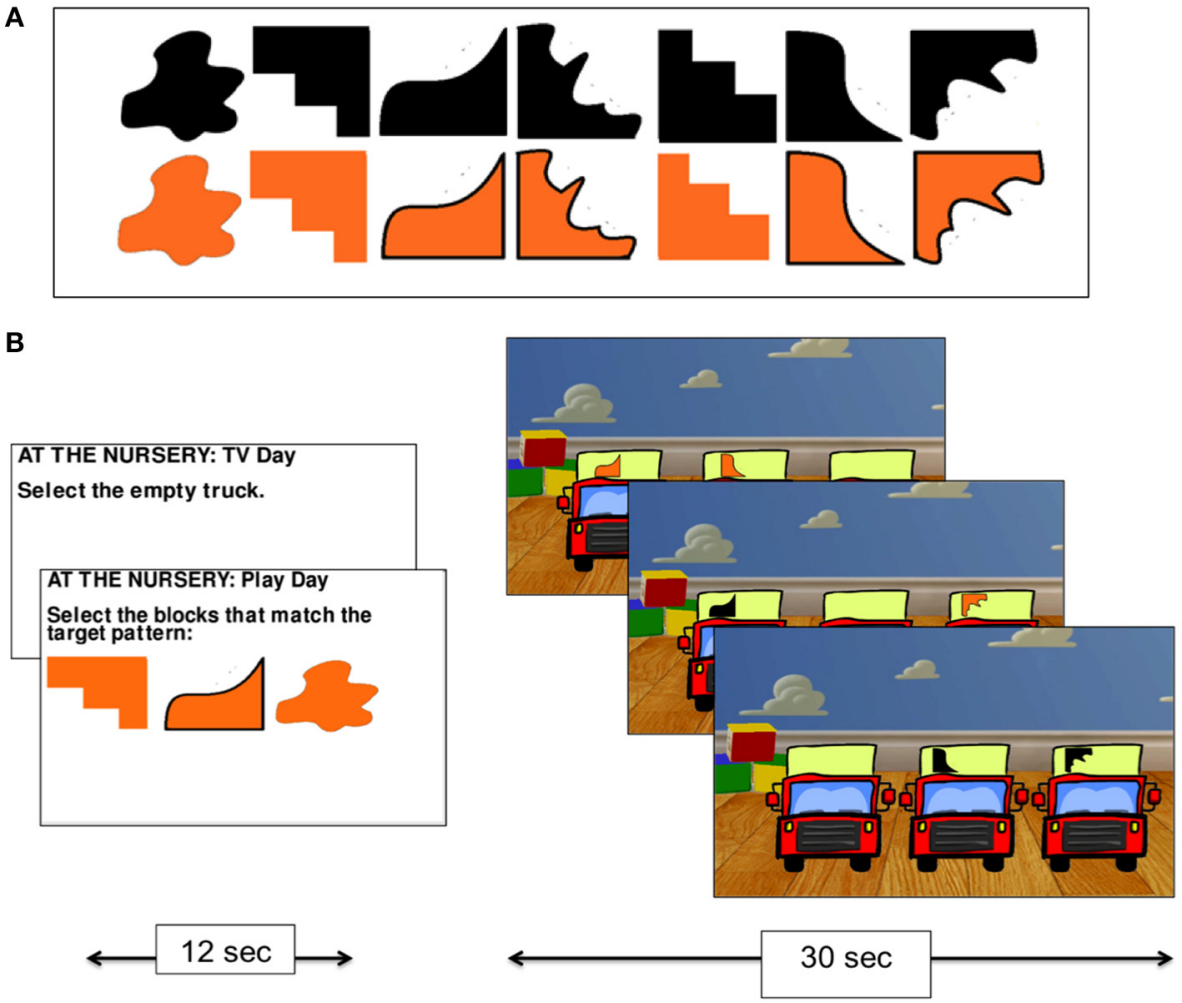
**The nursery game**. The set of shapes used on the trucks in the nursery game **(A)**, and experimental procedure **(B)**.

In the nursery game, participants were asked to imagine themselves as an infant in a nursery being presented with three trucks to play with. On each trial, they were asked to select which of the three trucks appearing on the screen they wished to play with. There were always two trucks marked with a randomly selected shape representing toy blocks, and one truck that was not marked with a shape—the “empty truck” (Figure 2B).

The nursery game had two conditions. In the *no monitoring condition* (Condition 1), participants were told that they preferred to watch TV rather than play with the trucks and so were instructed to always select the empty truck. This condition was used as a control task for subtracting visual processing and mouse clicking from the monitoring condition. In the *monitoring condition* (Condition 2), participants were told that they wanted to play with the trucks and that their goal was to collect the blocks (by selecting the relevant truck holding that shape using the mouse) that were required to match a pattern of three shapes that was presented at the start of the condition (the shapes were drawn from the set presented in Figure 2A). While no constraints were placed on the order with which participants collected the shapes in Condition 2, participants were given a reference pattern to work toward. The task, therefore, required participants to monitor their progress over time by keeping track of which shapes they had collected and which they still needed to collect.

In both games the vehicles were animated to move toward the participant for 1.5 s before the participant was able to make a selection. Two aspects of participants' performance were measured during the tasks. First, we recorded which vehicle participants selected on each trial. This information was used to verify that participants followed the instructions. Responses were scored by assigning one point every time a participant selected an appropriate vehicle (e.g., selected a tourist boat in the no monitoring condition of the harbormaster game), and zero points if they selected the wrong vehicle (e.g., selected a tourist boat on a trading day). Second, we recorded participants' responses to the questions designed to check that participants were monitoring progress as required in the harbormaster game.

### Procedure

Participants were given instructions for each game and an opportunity to practice all conditions on a computer before entering the scanner. Once lying in the scanner, participants practiced the games a second time to familiarize themselves with the MRI compatible mouse. Stimuli were viewed on a LCD screen via a head-coil-mounted, rear-facing mirror.

### Scan design

**Figure 3:**
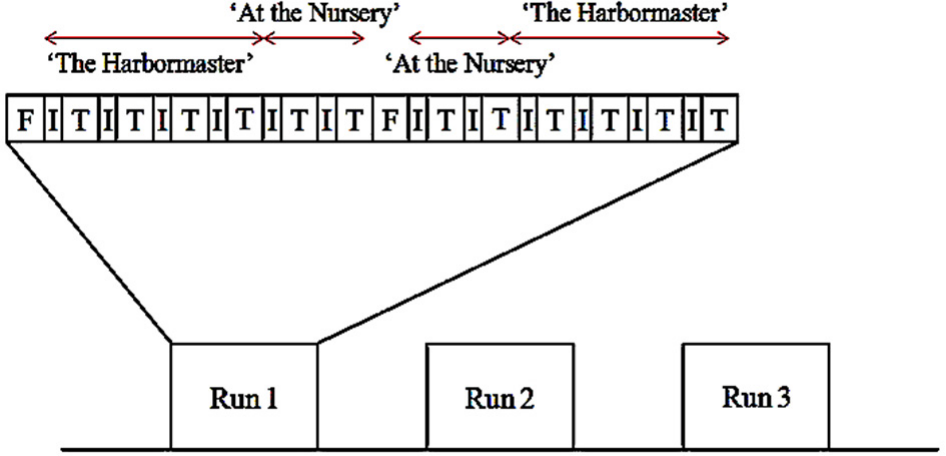
**Example of the scan design (F, fixation block; I, instruction block; T, trial block)**.

The experiment used a block design, with three runs (Figure 3). Each run began with a 30-s fixation block. Thereafter, participants performed each condition for 30 s preceded by instructions for 12 s. The conditions of each game were grouped (i.e., participants played four conditions of the harbormaster game before playing two conditions of the nursery game). The order of the games, as well as the order of the conditions within each game, was counterbalanced between and within participants. Once participants had completed the first set of two games, a 30 s fixation period provided a short break before starting the next set of two games. Following the functional scans, a high-resolution, whole-brain 3D structural scan was acquired. In total, participants played each game six times and were in the scanner for approximately 50 min.

### Data acquisition

All MR images were acquired at 3T (Ingenia 3.0T, Philips Healthcare, Best Holland) using a fifteen-channel radiofrequency receive-only head coil. Cerebral vascular response to the tasks was recorded using the blood oxygenation level-dependent (BOLD) T2^*^-weighted signal time-course. During each functional scan, a time series of 194 dynamic datasets was obtained using a 2-dimensional single-shot, echo-planar imaging (EPI) sequence. The EPI scan parameters were as follows: repetition time (TR) = 3000 ms; echo time (TE) = 35 ms; sensitivity-encoding factor = 1.8; flip angle = 90°; in-plane voxel size = 2.4 × 2.4 mm interpolated to 1.8 × 1.8 mm; 35 contiguous 2-dimensional transaxial slices each having slice thickness = 4 mm. Anatomical reference data were obtained for each subject using a Magnetization Prepared-Rapid Acquisition Gradient Echo (MP-RAGE) technique (TE = 3.8 ms; TR = 8.3 ms; TI = 963 ms; flip angle = 8°). This 3D-encoded acquisition yielded T1-weighted data covering the entire intra-cranial structures at a voxel resolution of 1 × 1 × 1 mm.

### Data processing

Data analysis was performed using SPM8 (Wellcome Department of Imaging Neuroscience, London; www.fil.ion.ucl.ac.uk/spm/) implemented in MatLab (The MathWorks Inc., Natick, MA). Functional images were corrected for spatial variation between dynamics. Realigned images were then spatially normalized to the standard SPM EPI template. Following normalization, images were smoothed using an 8 mm full-width half-maximum (FWHM) Gaussian filter. After specifying the block-design matrix for each participant, the BOLD signals obtained under different conditions were assessed using a general linear model. Statistical contrasts were constructed for each individual, and then used for the second level group analysis. All contrasts presented here were created using a Family Wise Error (FWE) correction (*p* < 0.05), unless otherwise stated.

## Results

### Behavioral responses

**Figure 4:**
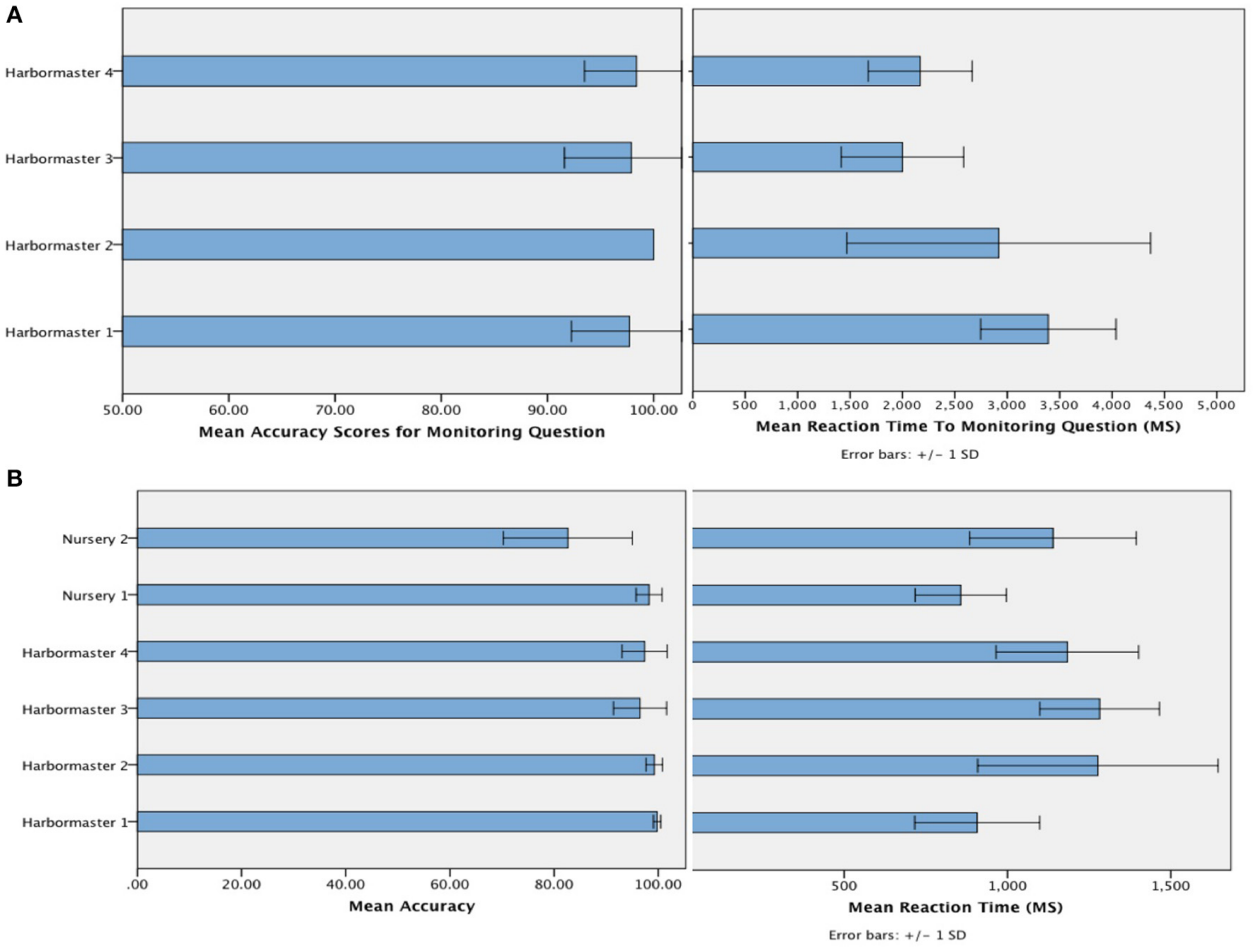
**Mean accuracy and reaction time to the question designed to check that participants were monitoring as intended in the harbormaster game (A) and mean accuracy and reaction time of responses (i.e., target selection) across both games (B)**.

The mean accuracy with which participants selected the targets (Figure 4) suggests that participants followed the instructions, and were reasonably competent in doing so (minimum of 80% accuracy)^1^. Given that chance level on each trial is 33%, and the average number of trials per block was 7, this level of accuracy is likely to be achieved by chance in just 0.08% of cases. In terms of the time taken to respond to the questions designed to assess monitoring, a One-Way ANOVA revealed a significant difference between the conditions of the harbormaster game, *F*_(3, 75)_ = 11.52, *p* < 0.001. Participants took longer to answer the simple addition questions in Condition 1, which involved no monitoring, compared to the questions about monitoring asked in Condition 2 (Mean difference = −370.49 ms, *p* < 0.001), Condition 3 (Mean difference = −376.84 ms, *p* < 0.001), and Condition 4 (Mean difference = −284.53 ms, *p* = 0.003). This further suggests that participants followed the task instructions because in Condition 1 participants needed to perform the relevant calculation in response to the question as it was presented, whereas participants in the monitoring conditions were monitoring and updating the relevant information as they went along and so did not need to make any new calculations in response to the question.

### Imaging results

Given that all conditions had a high rate of correct responses, as reflected by the behavioral data, we did not exclude individual responses from the fMRI analysis as they are unlikely to significantly influence the group data. Furthermore, since a similar level of errors was observed in all conditions, it is likely that activations related to errors would not be statistically significant following subtraction of one condition from another.

#### The harbormaster game

There were four conditions in the harbormaster game: no monitoring (Condition 1), trial-by-trial monitoring (Condition 2), monitoring over-time without a reference value (Condition 3), and monitoring progress over-time with respect to a reference value (Condition 4). To examine the neural processes involved in the different types of monitoring, we computed three contrasts: Condition 2 > 1, Condition 3 > 1, and Condition 4 > 1. Contrast 2 > 1 involved right superior parietal, left inferior parietal and bilateral cingulate and superior and medial frontal gyri. Contrast 3 > 1 resulted in activation in the right middle and medial frontal gyri, cingulate gyrus and left inferior and superior parietal regions. Contrast 4 > 1 resulted in activation of the bilateral precuneus, inferior and superior parietal lobules, inferior, medial and middle frontal gyri and cingulate gyrus (Table 1). The dACC was only significantly activated in the contrast between Conditions 4 > 1.

**Table 1:** **Brain regions activated in the different conditions of the harbormaster game (FWE Corrected, *p* = 0.05)**.

**Region**		**BA**	**Max coordinate**			**Z score**	**Cluster size**	**Beta value**
**Lobe**	**Anatomical localization of cluster**		***X***	***Y***	***Z***			
**CONDITION 2 > CONDITION 1**								
**Right**								
Parietal	Superior parietal lobule	–	30	−64	46	4.87	1	1.03
**Inter-hemispheric**								
Frontal	Superior (L,C,R)/medial (L,R) frontal gyrus	6(L,R),8(L)	0	12	56	5.35	19	1.38
Frontal(L)/limbic(L,R)	Cingulate gyrus	32	−8	18	42	5.36	27	1.16
**Left**								
Parietal	Inferior parietal lobule	40	−34	−54	48	5.25	12	1.29
	Inferior parietal lobule	40	−44	−40	46	5.32	25	1.54
Frontal	Inferior frontal gyrus	–	−48	10	20	5.0	1	1.47
**CONDITION 3 > CONDITION 1**								
**Right**								
Frontal	Middle frontal gyrus	8	32	22	52	5.28	21	2.0
Frontal/limbic	Cingulate/medial frontal gyrus	32	4	24	42	6.52	213	1.91
Parietal	Inferior parietal lobule	40	50	−36	48	5.39	76	1.51
**Left**								
Parietal	Inferior parietal lobule	40	−6	−74	50	5.39	76	1.65
	Precuneus/superior parietal lobule	7	50	−36	48	5.92	105	1.51
**CONDITION 4 > CONDITION 1**								
**Right**								
Parietal	Inferior parietal lobule, sub gyral	–	34	−60	40	4.97	3	1.2
	Inferior parietal lobule, post-central gyrus	40	50	−38	48	5.53	97	1.35
	Inferior parietal lobule	7	36	−62	44	4.97	2	1.33
	inferior parietal lobule	40	48	−60	46	4.98	3	1.09
	Inferior/superior parietal lobule	7	36	−68	48	5.13	16	1.44
	Precuneus, superior parietal lobule	7	8	−68	48	5.64	77	1.68
Frontal	Insula/inferior frontal gyrus/extra nuclear	47	34	18	−6	5.57	19	0.99
	Middle frontal gyrus	–	−44	46	−4	5.26	9	1.5
Frontal/limbic	Inferior/medial/middle/superior/cingulate frontal gyrus	6,8,9,46	38	34	32	6.37	758	1.6
Limbic	Anterior cingulate	–	10	32	26	5.01	1	1.03
**Left**								
Cerebellum	Posterior lobe-Uvula	–	−32	−64	−34	5.0	4	1.25
Parietal	Precuneus, inferior/superior parietal lobule	7,19,40	−44	−40	44	6.45	396	1.59
	Precuneus	7	−8	−72	48	5.63	29	1.27
Frontal	Inferior/middle frontal gyrus	10,46	−42	38	18	5.17	37	1.8
	Middle/-18superior frontal gyrus	6	−18	14	58	4.98	7	1.12
Frontal/limbic	Cingulate/medial frontal gyrus	8,32	−2	20	48	5.36	22	1.76

To identify the differences between monitoring over time and trial-by-trial monitoring, we computed the contrast between Conditions 3 > 2, where WM and calculation demands are similar. The results revealed large areas of activation in the right superior and middle frontal gyri (BA8/9/10) (Table 2).

**Table 2:** **Brain regions identified for monitoring over time (Condition 3) relative to trial-by-trial monitoring (Condition 2) in the harbormaster game (FWE Corrected, *p* = 0.05)**.

**Region**		**BA**	**Max coordinate**			**Z score**	**Cluster size**	**Beta value**
**Lobe**	**Anatomical localization of cluster**		***X***	***Y***	***Z***			
**HARBORMASTER 3 > HARBORMASTER 2**								
**Right**								
Sub-lobar	Insula/extra nuclear	13	32	14	−6	5.14	2	0.77
	Extra nuclear	13	30	16	−8	4.86	1	0.65
		–	20	14	10	4.91	1	0.45
		–	8	−4	0	5.28	5	0.5
Frontal	Middle frontal gyrus	10	40	50	8	5.04	15	1.54
	Superior frontal gyrus	10	30	56	20	4.91	3	1.59
	Superior/middle frontal gyri	8/9/10	22	32	42	5.97	386	1.17
Limbic/parietal	Cingulate gyrus/precuneus	7	6	−36	44	4.86	3	0.9
**Left**								
Frontal	Middle frontal gyrus	8	−24	24	50	4.97	1	0.83
	Superior frontal gyrus	6	−20	12	60	4.88	1	0.84

To further examine the neural basis of monitoring progress over time with a clear reference value, we computed the contrasts from BOLD imaging data between Conditions 4 > 2 and Conditions 4 > 3 (Table 3). The contrast between Conditions 4 > 2 revealed activations of the right middle frontal gyrus (BA9) and inferior parietal lobule (BA39, BA40). The contrast between Condition 4 > 3 differed only on one dimension—whether monitoring occurred with or without a reference value or goal—and revealed only one area of activation in the left cuneus (BA17) (Table 3).

**Table 3:** **Brain regions identified for monitoring over time with respect to a reference value (Condition 4) vs. monitoring over time without a reference value (Condition 3) and trial-by-trial monitoring (Condition 2) in the harbormaster game (FWE Corrected, *p* = 0.05)**.

**Region**		**BA**	**Max coordinate**			**Z score**	**Cluster size**	**Beta value**
**Lobe**	**Anatomical localization of cluster**		***X***	***Y***	***Z***			
**HARBORMASTER 4 > HARBORMASTER 2**								
**Right**								
Parietal	Inferior parietal lobule	40	52	−44	46	5.24	11	1.21
	Angular gyrus/inferior parietal lobule	39,40	48	−64	38	5.04	10	1.21
Frontal	Middle frontal gyrus	10,46	44	44	18	5.10	7	1.73
		–	26	18	44	4.89	1	1.19
	Middle/superior frontal gyrus	9	38	38	34	5.81	35	1.49
**HARBORMASTER 4 > HARBORMASTER 3**								
**Left**								
Occipital	Cuneus	17	−16	−88	4	5.37	26	1.01

#### The nursery game

The nursery game was designed to examine the brain regions involved in monitoring progress toward a visuo-spatial target. To do so, we computed the contrast between Conditions 2 > 1 (Table 4). This contrast is conceptually similar to the contrast between Conditions 4 > 1 in the harbormaster game as both compare monitoring progress over time with a reference value to no monitoring. Much like the contrast between Conditions 4 > 1, this contrast resulted in an extensive fronto-parietal network of activation. However, while in the contrast for the nursery game this network was bilateral, the contrast between Conditions 4 > 1 of the harbormaster game predominantly involved the left parietal and right frontal regions. In addition, the contrast between Conditions 2 > 1 of the nursery game included the dACC and bilateral cuneus (BA17).

**Table 4:** **Brain regions identified for monitoring progress (Condition 2) vs. not monitoring progress (Condition 1) in the nursery game**.

**Region**		**BA**	**Max coordinate (MNI)**			**Z score**	**Cluster size**	**Beta value**
**Lobe**	**Anatomical localization of cluster**		***X***	***Y***	***Z***			
**RIGHT**								
Cerebellum	Posterior lobe/Pyramis	–	24	−64	−38	5.27	7	0.90
Occipital	Cuneus	17/28/23/30	12	−74	10	5.4	76	1.38
		18	6	−90	18	4.99	6	1.11
Parietal	Inferior/superior parietal lobule/precuneus/sub gyral	7/19/39/40	32	−72	44	6.01	301	2.21
Frontal	Inferior frontal gyrus	47	30	24	−6	5.14	43	2.48
	Inferior frontal gyrus/sub-gyral	–	46	10	18	4.95	6	2.31
	Middle frontal gyrus	10	36	50	6	4.97	3	2.20
		9/46	48	32	30	5.45	100	3.17
	Medial frontal gyrus	–	10	36	36	4.84	1	1.37
Thalamus	Pulvinar	–	20	−30	2	5.02	1	0.49
		–	22	−28	4	4.85	1	0.48
Limbic	Anterior cingulate/cingulate gyrus	–	12	26	30	4.95	2	1.19
**INTER-HEMISPHERIC**								
Parietal	Precuneus (L,R)/sub-gyral (R)/superior parietal (L)	7	10	−76	46	5.82	200	1.64
Limbic	Cingulate gyrus/medial frontal gyrus (L,R)	6,8,32 (L,R)	4	24	46	6.17	239	2.66
**LEFT**								
Cerebellum	Anterior lobe/Culmen	–	−38	−44	−26	4.84	1	1.19
Occipital	Cuneus	17	−12	−78	4	5.28	33	1.29
		18/19	−12	−90	22	5.18	19	0.84
Occipito-temporal	Fusiform gyrus/sub-gyral	37	−46	−60	−14	5.09	14	2.0
Parietal	Inferior/superior parietal lobule/sub gyral	7/19/40	−28	−68	42	6.08	317	2.71
	Inferior parietal lobule	–	−46	−38	44	4.98	6	1.58
Frontal	Insula/inferior frontal gyrus	13/47	−30	20	−6	5.19	21	2.0
	Middle/inferior frontal gyrus/sub-gyral	46	−46	32	22	5.66	168	2.92
	Inferior frontal gyrus/sub-gyral	–	−40	8	24	5.11	20	2.64
	Middle frontal gyrus	6	−28	10	62	5.71	18	1.28
Thalamus	Pulvinar	–	−10	−8	−2	4.96	1	0.52
Sub-lobar	Extra nuclear/corpus callosum	–	−2	4	24	4.86	3	0.93

*FWE Corrected, p = 0.05*.

#### Conjunction (inclusive) analysis of the harbormaster and nursery games

**Figure 5:**
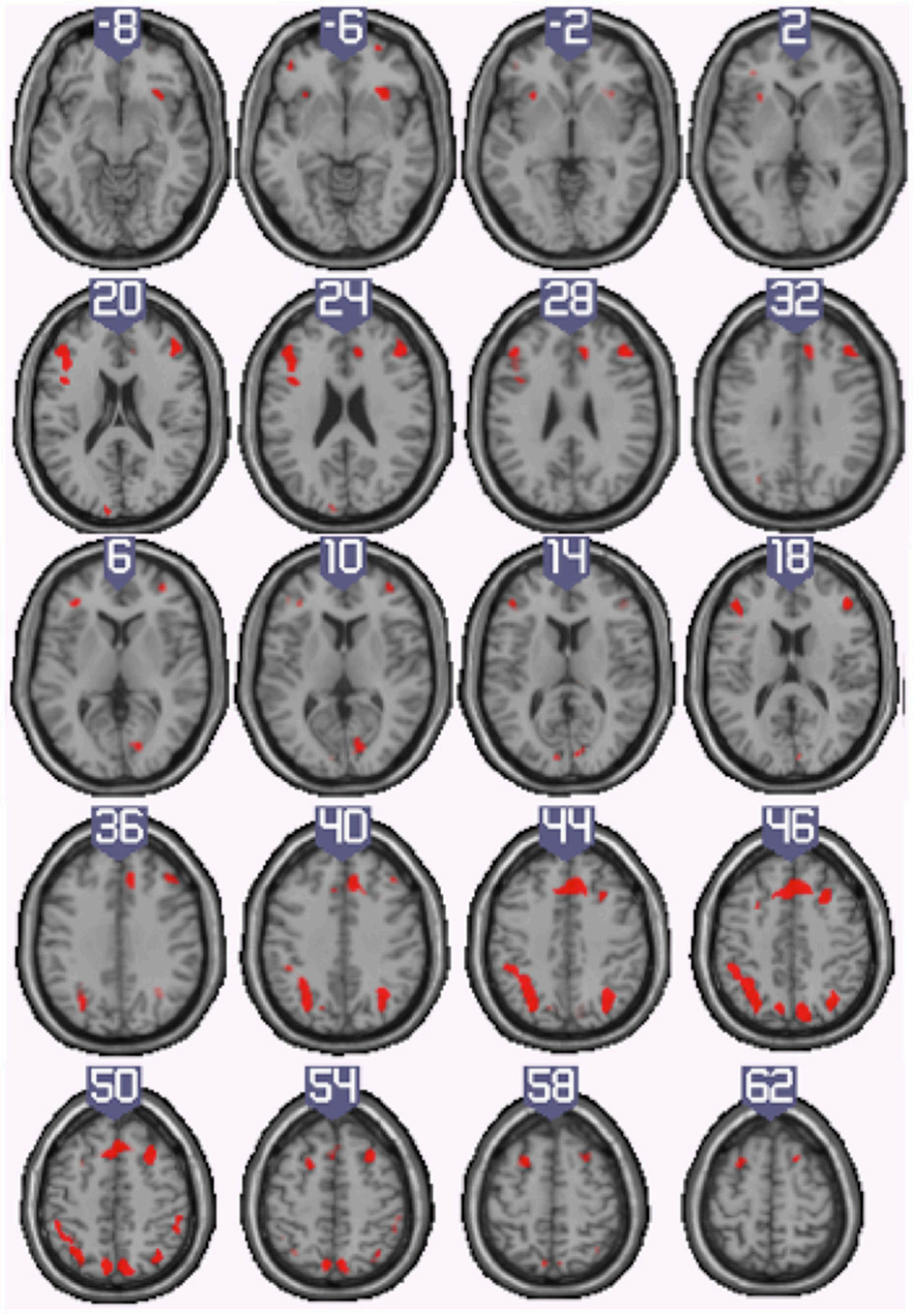
**Illustration of the neural regions activated by the conjunction (inclusive) between the harbormaster (4 > 1) and nursery (2 > 1) games**. (FWE corrected, *p* = 0.05, *K* = 0).

To identify the neural basis of progress monitoring in a way that is relatively independent of the modality of the target representations (verbal or visual), we computed the combined contrast between Conditions 4 > 1 of the harbormaster game and Conditions 2 > 1 of the nursery game. The results are presented in Table 5 and Figure 5. As expected from the activations observed in the two games individually, an extended fronto-parietal network was activated when monitoring progress over time with respect to a reference value. Activations were also observed in the bilateral primary visual cortex (BA 17/18) and the dACC.

**Table 5:** **Brain regions activated during monitoring progress over time with respect to a reference value in both the harbormaster and the nursery games (FWE corrected, *p* = 0.05)**.

**Region**		**BA**	**Max coordinate**			**Z score**	**Cluster size**	**Beta value**
**Lobe**	**Anatomical localization of cluster**		***X***	***Y***	***Z***			
**RIGHT**								
Occipital	Cuneus	17,18,23,30	14	−78	6	5.21	73	1.32
Parietal	Inferior/superior parietal lobule, precuneus, angular gyrus, sub-gyral	7,19,39,40	32	−70	44	6.22	256	2.62
	Superior parietal lobule, precuneus	7	10	−78	48	5.82	178	2.92
	Inferior parietal lobule, post-central gyrus	40	52	−44	50	5.83	44	1.86
Frontal	Inferior frontal gyrus. Insula, sub-gyral	13,47	34	20	−6	5.32	74	2.85
	Middle/superior fontal gyrus	10	28	60	−8	5.31	11	3.73
	Middle frontal gyrus, sub-gyral	10	36	50	6	5.44	29	3.61
	Inferior/middle/superior frontal gyrus	9,10,46	42	34	34	5.71	233	4.26
	Middle/superior frontal gyrus	6,8	30	20	46	6.35	188	2.43
**INTER-HEMISPHERIC**								
Limbic/frontal	Anterior cingulate(R), cingulate/medial frontal gyrus(L,R), superior frontal gyrus (L,C,R)	6,8,32(L,R),9(R)	6	26	46	6.78	530	2.66
**LEFT**								
Cerebellum	Posterior lobe-cerebellar tonsil	–	−38	−58	−54	4.93	8	1.7
Occipital	Cuneus	18	−10	−88	12	5.09	8	1.17
		18,19	−8	−94	22	5.2	20	1.11
Parietal	Inferior/superior parietal lobule, precuneus, supramarginal/angular gyrus, sub-gyral	7,19,39,40	−28	−68	42	6.49	588	2.72
	Precuneus, superior parietal lobule	7	−6	−74	50	6.25	119	2.17
Frontal	Inferior frontal gyrus/insula/extra nuclear	13,47	−28	20	0	5.25	29	1.73
	Inferior frontal gyrus, sub-gyral	–	−36	38	6	5.5	29	1.08
			−40	10	24	5.29	55	2.14
	Middle frontal gyrus	–	−42	44	−6	5.52	13	2.62
		6	−26	12	46	5.01	10	1.38
	Inferior/middle frontal gyrus, sub-gyral	46	−46	34	22	5.82	238	3.4
	Middle/superior/medial frontal gyrus	6,32	−24	14	60	5.52	76	1.63

## Discussion

The present research examined the neural regions involved in monitoring goal progress, across numerical and visuo-spatial modalities. Two computer games were designed whose conditions differed in the nature of the progress monitoring that was required. In the harbormaster game, Condition 1 acted as a baseline condition, Condition 2 involved trial-by-trial monitoring, Condition 3 involved monitoring information over time without comparing progress to a reference value, and Condition 4 involved monitoring progress over time with respect to a numerical reference value. In the nursery game, Condition 1 acted as a baseline condition, while Condition 2 involved monitoring progress over time with respect to a reference value of a visual nature. The findings from both games were used to identify a modality-independent network of activations involved in monitoring progress over time.

The findings point to a series of activations in the fronto-parietal network, including the right DLPFC (BA9) and bilateral inferior and superior parietal regions. This network is largely similar to activations identified in studies of the neural basis of attention and WM (Corbetta and Shulman, 2002; Fox et al., 2005; Gazzaley and Nobre, 2012) supporting the idea that these processes are involved in monitoring goal progress. In addition, the dACC was activated when progress was measured against a reference value confirming previous findings, which indicate that the dACC is activated in response to situations where a discrepancy between the current and target state is likely (Carter et al., 1998; Ullsperger and von Cramon, 2001; Kerns et al., 2004; Abutalebi et al., 2012; Berkman et al., 2012). Finally, despite identical visual stimuli being presented in the monitoring and no monitoring conditions, monitoring with respect to a reference value resulted in activation of early visual processing regions (BA17). We suggest that this activation provides evidence of top–down processing of goal-directed attention to information in the primary visual cortex (Chawla et al., 1999; Hopfinger et al., 2000).

To our knowledge, the present research is the first to examine the neural basis of monitoring progress over a medium term, rather than on a trial-by-trial basis. As predicted, all conditions (in comparison to baseline) increased activation in the fronto-parietal network. This included activation of the inferior frontal gyrus, previously shown to be involved in stimulus-driven attention (Corbetta and Shulman, 2002) and the right superior parietal cortex and superior and middle frontal gyri (BA9, BA10), including the frontal eye field, which have previously been implicated in goal-directed attention (Corbetta and Shulman, 2002; Fox et al., 2005). A similar network has been shown to be involved in WM (Gazzaley and Nobre, 2012). This pattern of activation, suggests that attention and WM resources may be needed for the effective monitoring of goal progress.

We hypothesized that the DLPFC would be activated when monitoring goal progress over time, due to its involvement in updating WM (Petrides, 2000; Provost et al., 2010). Consistent with this idea, two of the contrasts in the harbormaster game (contrasts 4 > 2 and 3 > 2) and the contrast in the nursery game (contrast 2 > 1), revealed activations in the right DLPFC (BA9). These findings are likely to reflect the fact that these conditions required participants to keep track of their current state and update this value on each trial (e.g., when fish were bought or sold, or new shapes were added to the current collection). We conclude, therefore, that the right DLPFC is likely to play an important role in monitoring progress toward goals over time, regardless of the nature of information that needs to be considered.

Activations in the inferior and superior parietal cortices were also observed in all conditions involving monitoring (e.g., trial-by-trial and over time) compared with the baseline conditions. It is likely that in some contrasts (namely, contrasts 2 > 1, 3 > 1 and 4 > 1 in the harbormaster game, contrast 2 > 1 in the nursery game) activations of the right superior and left inferior parietal reflect, at least in part, processes involved in calculation (Benn et al., 2012) and the manipulation of visuo-spatial information (Culham and Kanwisher, 2001). However, parietal activation may also be directly related to the process of monitoring information over time since parietal activation was also observed in contrasts 4 > 2 and 3 > 2 of the harbormaster game (where calculation demands are similar) and in the nursery game contrast, where parietal activation extended over a large bilateral region including both inferior and superior parietal lobules. Several studies using fMRI (e.g., Rao et al., 2001; for a review, see Lewis and Miall, 2003) and EEG (Mohl and Pfurtscheller, 1991) show that the right parietal cortex, particularly the right inferior parietal region, plays a role in the perception of time. It has further been suggested that the right parietal cortex is a hub for the processing of magnitude, including numerical, spatial and temporal processing (Walsh, 2003). It therefore seems likely that, regardless of the modality of the stimuli being processed, a circuit involving the frontal-parietal network is involved in monitoring goal progress over time, as demonstrated by the joint contrast of the nursery and harbormaster games.

In contrast to the consistent activation observed in the fronto-parietal network, the dACC, which has been previously associated with discrepancy detection and reduction (Carter et al., 1998; Ullsperger and von Cramon, 2001; Kerns et al., 2004; Abutalebi et al., 2012; Berkman et al., 2012), was only activated in the contrasts where the baseline condition was compared to a task that involved monitoring with respect to a reference value. In the present paradigm, the dACC was likely involved in identifying potential discrepancies between the current state (i.e., the amount of fish in the port or the current combination of shapes that had been collected) and the reference value (i.e., the total amount of fish allowed at the port, or the target set of shapes). These findings suggest that, while the dACC is an important part of the process of monitoring progress, it is not the only process involved, and monitoring progress over time often involves attending to and updating information without any discrepancy necessarily occurring (e.g., when progress is as expected).

As noted earlier, monitoring with respect to a reference value (contrast 4 > 3 of the harbormaster game and the contrast between the conditions of the nursery game) led to activation in early visual processing regions (BA17). This finding is consistent with accumulating evidence that early sensory processing is involved in goal-directed behavior. For example, evidence suggests that top-down directed attention results in increased firing of neurons in the visual cortex (V1 and V4) of rhesus monkeys (McAdams and Maunsell, 1999), and that goal-directed attention can modulate activity in the early visual cortex of humans (Chawla et al., 1999; Hopfinger et al., 2000). More recently, Levita et al. (2014) demonstrated using EEG that the early visual cortex is involved in processing learned danger signals (i.e., serving the goal of avoiding harm). In the current research, while the two tasks involving monitoring progress with respect to a reference value evoked activation in this region, this activation was more pronounced in the nursery game, where the reference value and the expected stimuli were of visual nature (i.e., not nameable). Future research might, therefore, investigate how the nature of the reference value as well as the nature of the to-be-monitored information influences activations in early sensory cortices.

## Limitations and future directions

Monitoring strategies may differ between individuals. For example, one participant may keep track of the shapes that they have collected by trying to form an aggregated image, while another participant may try to remember individual shapes. However, in the present research we analyzed the findings at the group level, assuming that all participants approached the task in a similar way—which seemed reasonable given the relatively constrained nature of the focal tasks. Future research might, however, wish to examine the neural basis of different monitoring strategies. This could be done in a quasi-experimental fashion by asking participants what strategy (or strategies) they used, or in an experimental fashion by directing participants to use one strategy or another. Relatedly, the behavioral responses in the present experiment (namely, whether participants selected appropriate targets, or could report on the relevant dimension that they were monitoring) were simply designed to ensure that participants followed task instructions, rather than to elucidate the nature or difficulty of monitoring progress. Future research could, however, use behavioral measures to examine the nature of progress monitoring—e.g., examining the effect of different monitoring strategies on performance on a secondary task (e.g., one involving WM). We would expect that more demanding forms of monitoring, such as comparing a current value to a reference value, would impact performance on a secondary task to a greater extent than less demanding forms of monitoring.

In addition, given the role of the parietal cortex in both numerical and visuo-spatial processing and the observed activation in primary visual cortex, one limitation of the present research is that it is difficult to identify the determinants of activations in these regions. Future research could, however, address this issue by using an auditory paradigm. For example, participants could work toward recreating a musical rhythm by selecting sub-patterns of this rhythm. An auditory paradigm could further help to establish whether monitoring goal progress affects activation in other primary sensory cortices, and to what degree parietal activation can be attributed to visuo-spatial, numerical or attentional processing.

## Conclusion

The present research investigated the neural correlates of monitoring goal progress over a medium-term period. Our findings largely support the view that the dACC plays a role in discrepancy detection, which is an important aspect of progress monitoring. However, the present research also helped to identify the neural basis of monitoring progress over time, something that has been relatively neglected in previous studies. We found that regions of the parietal cortex, as well as the right DLPFC, are involved in monitoring progress over time—something that is likely due to monitoring placing demands on attention and WM resources as well the requirement for checking and updating of information over time. Lastly, we report evidence that progress monitoring activated regions of the primary visual cortex, adding to growing evidence that the primary sensory cortices may play an important role in monitoring information in relation to specific goals.

### Conflict of interest statement

The authors declare that the research was conducted in the absence of any commercial or financial relationships that could be construed as a potential conflict of interest.

## Footnotes

^1^On participant was identified whose performance was significantly worse than the average for the group in two out of the six conditions (>2.5 standard deviations from the mean). To see whether inclusion of this participant influenced our findings, we reran the behavioral and fMRI analysis excluding this participant. The results were largely unchanged and so the analyses reported include all participants.
